# Geospatial mapping and 7-year temporal trends of electromagnetic field bands in Cyprus

**DOI:** 10.1007/s10661-026-15361-7

**Published:** 2026-05-07

**Authors:** Yiannis Kiouvrekis, Ioannis Psomadakis, Christos Christakis, Dimitris Kalatzis

**Affiliations:** 1 Mathematics, Computer Science and Artificial Intelligence Laboratory, University of Thessaly, V.Griva 13, Karditsa, 43131 Thessaly Greece; 2 Department of Information Technologies, University of Limassol, 46 Makedonitissas Avenue, Limassol, 3020 Cyprus; 3 Business School, University of Nicosia, 46 Makedonitissas Avenue, Nicosia, 2417 Cyprus; 4 Department of Environmental Sciences, University of Thessaly, Larissa, 41500 Thessaly Greece

**Keywords:** Electromagnetic fields (EMF), Radiofrequency exposure, Geospatial mapping, Spatiotemporal analysis, Environmental monitoring, 5 G networks, Cyprus, Temporal trends, Public health, EMF regulation

## Abstract

This study presents the first integrated geospatial and temporal assessment of radiofrequency electromagnetic field (RF-EMF) exposure in Cyprus, using 7 years (2017–2023) of periodic in-situ measurements conducted at fixed locations around all operational mobile telephony base stations as part of the national RF-EMF monitoring program. Electric field strengths were evaluated across eleven frequency bands spanning 30 MHz–3.6 GHz, including broadcast services and cellular communication bands relevant to 4 G and 5 G networks. Spatial exposure distributions were characterized through geostatistical interpolation, while long-term variability was quantified using non-parametric Kruskal-Wallis and Mann–Kendall tests. Results show that exposure levels in all bands remain well below international reference limits. Broadcast bands exhibit consistently low and stable values (< 1 in µV/m), whereas significant increasing monotonic trends were detected in several mobile communication bands, particularly 800 MHz, 1800 MHz, 2100 MHz, and 2600 MHz, reflecting network densification and growing data demand. The newly introduced 700 MHz and 3600 MHz 5 G bands did not yet display statistically significant trends due to the shorter observation period. The combined spatiotemporal evidence highlights localized hotspots in high-traffic areas and underscores the need for sustained, transparent monitoring to ensure safe and adaptive EMF exposure governance in the evolving wireless landscape.

## An introduction

**Table 1:** Reference levels for general public exposure to time-varying electric and magnetic fields (unperturbed rms values)

Frequency range	E-field strength	H-field strength	B-field	Equivalent plane wave
	(V m$^{-1}$)	(A m$^{-1}$)	(µT)	power density $S_{eq}$ (W m$^{-2}$)
up to 1 Hz	-	$3.2 \times 10^4$	$4 \times 10^4$	-
1–8 Hz	10,000	$3.2 \times 10^4/f^2$	$4 \times 10^4/f^2$	-
8–25 Hz	10,000	4000/*f*	5000/*f*	-
0.025–0.8 kHz	250/*f*	4/*f*	5/*f*	-
0.8–3 kHz	250/*f*	5	6.25	-
3–150 kHz	87	5	6.25	-
0.15–1 MHz	87	0.73/*f*	0.92/*f*	-
1–10 MHz	$87/f^{1/2}$	0.73/*f*	0.92/*f*	-
10–400 MHz	28	0.073	0.092	2
400–2000 MHz	$1.375f^{1/2}$	$0.0037f^{1/2}$	$0.0046f^{1/2}$	*f*/200
2–300 GHz	61	0.16	0.20	10

The general population is daily exposed to radio frequency (RF) electromagnetic fields (RF-EMFs) from various sources such as mobile phones, Wi-Fi routers, broadcast radio and television transmitters, and other wireless communication devices. The frequency band of RF-EMFs ranges from 3 kHz up to 300 GHz. Several recent studies have explored the potential adverse health effects of exposure to RF-EMFs in the 10 MHz to 6 GHz frequency range, generated by telecommunications base stations, mobile phones, Wi-Fi, wireless networks, TV broadcasting, AM/FM radio, etc. (Pophof et al., 2023). The impact of EMF exposure varies depending on the frequency and strength. Numerous epidemiological studies indicate correlations between RF-EMFs exposure and health effects such as cancer (Mead, 2008), neurological disorders (Kim et al., 2017), genetic effects (Lai, 2021), disturbances in the immune system (Piszczek et al., 2021), and other biological effects.

The International Commission on Non-Ionizing Radiation Protection (ICNIRP) has published Guidelines for protection against human exposure to EMFs in the 100 kHz-300 GHz range. The most recent ICNIRP Guidelines (2020) are based on computational simulation models which consider the anatomical models of the human body. According to ICNIRP Guidelines (International Commission on Non-Ionizing Radiation Protection (ICNIRP), 1998), the reference limits of electric strength (V/m) for general public exposure to EMFs in the 10 MHz to 6 GHz frequency range are 28 V/m for 10–400 MHz, 1.375 f1/2 V/m for *f* = 400–2000 MHz, and 61 V/m for 2–300 GHz (see Table 1).

According to the World Health Organization (WHO), so far, no biological mechanism has been established that can explain the association with adverse health effects. The available data are insufficient to definitively assess the associated health risks. However, the WHO demonstrates that even if there is no proven biological probability of such an association, its possible causal nature cannot be dismissed, nor should the magnitude of the risk be underestimated (World Health Organization, 2016). Precautionary approaches are recommended until the potential health effects of EMF are verified.

A large international case-control study in adults, the INTERPHONE study, did not show an overall increase in the risk of brain tumors with the use of the mobile phone (Group TIS, 2010). However, for long-term heavy use of mobile phones, the odds ratios were 1.4 for glioma and 1.15 for meningioma. In 2013, after the INTERPHONE study, the International Agency for Research on Cancer (IARC) reviewed epidemiological studies and classified RF-EMFs from mobile phones to GROUP 2B, as possibly carcinogenic to humans. Children are more vulnerable to RF exposure than adults, as a result of investigations on the effect of anatomical characteristics on the exposure of specific organs (Acharya et al., 2021; Jin-Hwa, 2020). The nervous system of children is more susceptible than that of adolescents (Kheifets et al., 2005). Their bone marrow has higher electric conductivity, so the exposure to RF can exceed a factor 10 in comparison with adults (Christ et al., 2010). Their brain regions (e.g., hippocampus, hypothalamus, cerebellum) exhibit higher local specific absorption rate (psSAR) than that of adults (Christ et al., 2006). Accordingly, public concern regarding electromagnetic exposure has increased (Ce et al., 2017) considering the simultaneous exposure to multiple frequency fields such as FM, VHF, UHF-TV, GSM900, UMTS900, UMTS1800, LTE800, LTE1800, LTE2100, LTE2600, 5 G-700, and 5 G-NR 3600.

In recent years, there has been increased reference to precautionary policies, and in particular to the Precautionary Principle, which is included in the World Health Organization’s report (World Health Organization, 2007). As a precautionary measure, some countries have reduced exposure limits. For example, in 2003, Italy adopted the ICNIRP standards but introduced two additional limits for exposure to electromagnetic fields: (i) “attention values” set at one-tenth of the ICNIRP reference levels for specific locations such as playgrounds, residences, and school facilities; (ii) stricter “quality objectives” that apply only to new sources and new residential buildings. The Precautionary Principle has also been mentioned in certain national legislations, for example, in Canada and Israel. The aim of this study was to provide a temporal analysis of electromagnetic field (EMF) bands in Cyprus using long-term data analyzed with statistical tools. The analysis seeks to map RF-EMFs, offering an overview of their distribution that is valuable for health risk assessment and the promotion of safe electromagnetic environments.

### Research objectives and contribution of the study

This study aims to provide a rigorous, policy-relevant cha-racterization of radiofrequency electromagnetic fields (RF-EMF) in Cyprus by integrating geospatial mapping with multi-year temporal analysis. We address the following research questions (RQs):

- **RQ1:** How do RF-EMF exposure levels vary spatially across Cyprus within distinct frequency bands (FM, VHF, UHF-TV, 700–3600 MHz)?
- **RQ2:** Do RF-EMF levels exhibit statistically signifi-cant temporal trends over the study period (2017–2023), and are these trends frequency-dependent?
- **RQ3:** Which bands and locations contribute most to upper-tail exposure (peaks/outliers), and how often do typical or peak values approach international reference levels?
- **RQ4:** What are the implications of the observed spatiotemporal patterns for environmental monitoring, public communication, and compliance with international exposure limits?

By explicitly linking spatial mapping with long-term exposure trends, this study introduces several innovative elements that advance the state of EMF exposure assessment beyond conventional measurement campaigns: (a) unlike most studies that focus on short-term measurements, this work presents a comprehensive 7-year (2017–2023) evaluation of EMF exposure trends in Cyprus in eleven distinct frequency bands, ranging from FM and VHF to modern cellular communication bands up to 3.6 GHz; (b) the combination of geospatial mapping and temporal trend analysis enables a detailed examination of both spatial distribution and long-term evolution of EMF exposure. This dual approach identifies persistent hotspots in urban regions and highlights post-2020 increases associated with the deployment of 5 G networks; (c) through the application of the nonparametric Kruskal-Wallis test, the study provides robust statistical evidence that EMF exposure variations are significantly influenced by both time and frequency band. (d) The outcomes offer valuable insights for policymakers, supporting the development of spatially resolved monitoring frameworks and adaptive safety guidelines aligned with ongoing technological evolution.

This study is based on data from Cyprus’ national RF-EMF monitoring program, which consists of standardized periodic in-situ measurements conducted at fixed locations around all operational mobile telephony base stations to assess ambient exposure at the population level. While such data do not capture individual exposure variability associated with indoor environments, personal mobility, or transport-related microenvironments, they offer a robust and authoritative basis for assessing long-term spatial and temporal exposure trends at the national scale. Personal exposure assessment would require complementary measurement approaches beyond the scope of the present work.

## Work in assessment of RF-EMF exposure in different environments

In recent years, several studies have reported RF-EMF exposure measurements in outdoor environments, focusing on public exposure near telecommunications sites. These investigations typically assess the contribution of mobile base stations, broadcast transmitters (FM, VHF, UHF-TV), and other RF sources to ambient electric field levels (Panagiotakopoulos et al., 2025; Beláčková et al., 2025). For example, country-specific surveys conducted in several countries have provided long-term datasets of outdoor exposure, allowing temporal trends and spatial variations to be evaluated (Kalatzis et al., 2025; Kiouvrekis et al., 2025). Such studies highlight that typical public exposure remains well below international reference limits, even in proximity to base stations, and provide valuable benchmarks for comparing measurement results across different countries. By including these references, the present work situates its findings within the broader context of outdoor RF-EMF exposure assessments and facilitates meaningful comparisons with previously published data.

Also, several recent studies have examined RF-EMF exposure levels in different environments around the world. Ramirez-Vazquez et al. (2023a) explored personal exposure levels to RF-EMF from the 2.4 GHz and 5.85 GHz Wi-Fi bands in a Spanish University. They found that the minimum average was 0.0900 $\mu$W/m2 in the 2.4 GHz Wi-Fi, while the maximum average reached 211 $\mu$W/m2 in the 5.85 GHz Wi-Fi across the campus. Intensity level maps were generated using the Kriging interpolation method. The same research group found that in Mexico City, the Wi-Fi exposure levels were 326 $\mu$W/m2 in the 2.4 to 2.5 GHz band and 2370 $\mu$W/m2 in the 5.15 to 5.85 GHz band (Ramirez-Vazquez et al., 2023b). In the school environment, two studies from Spain and Greece on Wi-Fi exposure and mobile networks showed that the exposure levels were well below the limit of the ICNIRP (International Commission on Non-Ionizing Radiation Protection) of 10 W/m2 for general public exposure (Alexias et al., 2020; Ramirez-Vazquez et al., 2020). In a similar study (Kiouvrekis et al., 2020) conducted in 492 primary and secondary Greek schools for frequencies between 27 MHz and 3 GHz, the mean electric field was measured at approximately 0.42 V/m. This value is lower than the RF limit set in Greece, which for sensitive areas is defined as 60% of those recommended by the European guidelines. In addition, Panagiotakopoulos et al. (2023) investigated RF-EMF exposure in Greek schools to support the spatial deployment of an IoT-based EMF monitoring system. Using stratified sampling across 661 schools in urban, semi-urban, and rural areas (27 MHz–3 GHz range), they reported a mean electric field strength of 0.42 V/m, well below both national and international safety limits. Interestingly, rural areas exhibited the highest peaks, indicating priority zones for future IoT EMF monitoring deployment.

Paniagua-Sánchez et al. (2023) presented an evaluation of human exposure to RF-EMF in a Spanish city using a personal exposure meter inside a moving vehicle. The authors reported that the mean electric field ranged from 0.057 to 0.231 V/m, depending on the location. A recent study by Manassas et al. (2025), which evaluated the exposure to EMF in urban and suburban areas in Greece during the deployment of 5 G, revealed two notable findings. The first is that the 4 G networks were the dominant contributors to overall EMF exposure levels, and the second is an inverse correlation between EMF levels and the distance from the corresponding 5 G base stations. Finally, a systematic review included a total of 86 studies from 1998 to 2023 with different methodologies (spot measurements, personal measurements, and predictive models) found that the lowest mean exposure was recorded in Palestine (0.060 $\mu$W/m2) and the highest in Norway (200,000 $\mu$W/m2). None of the reported studies exceeded the ICNIRP limits (Ramirez-Vazquez et al., 2024).

Villaescusa-Tebar and Garcia-Pardo (2025) examined 5 G-related RF-EMF exposure during a large public event in Valencia, Spain. Field measurements at 700 MHz and 3500 MHz showed that power density in the 3500 MHz band increased up to eightfold during the event compared to baseline levels, yet all values remained well below international safety limits. Longer (30 min) measurements revealed roughly 30% higher average exposure than 6 min samples, highlighting the impact of location and temporal variability on 5 G exposure assessments.

Deprez et al. (2025) evaluated 5 G radiofrequency electromagnetic field (RF-EMF) exposure across Belgium, Switzerland, Hungary, and Poland through 146 indoor and outdoor spot measurements conducted in 2023. Approximately 35% of sites established a 3.6 GHz 5 G connection. The highest measured power densities—17.6 mW/m$^{2}$ (No UE) and 23.3 mW/m$^{2}$ (Max DL)—were well below the ICNIRP safety limits. Exposure levels were higher in urban than in rural areas (ranging from −4.8 to −10.4 dB) and under line-of-sight (LOS) conditions (2.3 mW/m$^{2}$) compared to non-line-of-sight (NLOS) conditions (0.9 mW/m$^{2}$). Measurements in and around educational institutions were similar to those in public spaces, confirming that 5 G exposure levels remain low across all environments.

Iakovidis et al. (2025) analyzed 2 years of continuous EMF monitoring data (2022–2024) from 13 sensors across Greece’s major cities, focusing on the 3.6 GHz 5 G band. Results showed a gradual increase in EMF exposure linked to expanding 5 G infrastructure, with higher variability due to active antenna systems and traffic fluctuations. All measured values remained well below national and ICNIRP safety limits, highlighting the importance of continuous monitoring in the 5 G era. Djuric et al. (2025) analyzed long-term RF-EMF monitoring data from European systems and proposed a multi-scale time-averaging method to better assess temporal variability. Using a 5-year dataset from Serbia’s EMF RATEL network, the study demonstrated how this framework reveals daily and seasonal exposure patterns, offering improved insight into long-term EMF trends. Liu et al. (2025) analyzed uplink RF-EMF exposure from mobile devices across 2 G–5 G networks in the Greater Paris region. Measurements from 380 locations showed that 5 G devices emit lower transmit power and radiated energy per bit (5.1 mJ/Mb) compared to 4 G (9.65 mJ/Mb), indicating improved energy efficiency and reduced uplink exposure with 5 G technology.

Ruijie et al. (2025) assessed personal RF-EMF exposure in Malaysian micro-environments using the ExpoM-RF 4 m and machine learning models. The FCNN achieved the best prediction accuracy, and all exposure levels remained below international safety limits, with peaks near 56.7 V/m in dense urban areas. Chiaraviglio et al. (2025) proposed a narrow-band measurement method (EM-FWA) to compare EMF exposure from 5 G radio base stations (RBSs) and user terminals in Fixed Wireless Access networks. Results showed that terminal exposure can exceed RBS exposure at the cell edge (1.4 V/m vs. 0.2 V/m), highlighting the need for new EMF assessment approaches considering multiple active terminals. Pasquino et al. (2025) analyzed continuous EMF monitoring data from Serbia’s EMF RATEL network using a Log-Normal Mixture Model (LNMM). The model effectively distinguished day-night variations and workday-holiday patterns, demonstrating the usefulness of machine learning clustering methods for characterizing temporal EMF exposure trends.

A systematic review by Jalilian et al. (2019) analyzed RF-EMF levels across various European microenvironments, reporting mean outdoor exposure values ranging from 0.07 to 1.27 V/m, with mobile phone base station downlink identified as the primary contributor. Their findings indicated that while exposure levels remained well below regulatory limits, a clear gradient existed where RF-EMF intensity increased with urban density. Another systematic review by Ramirez-Vazquez et al. (2023a) evaluated personal RF-EMF exposure studies spanning over two decades (1998–2021) using PRISMA and PECO methodologies. Their analysis, which included 56 high-quality publications, highlighted that while exposure levels across various European and global microenvironments vary, ranging from negligible values in early studies to peaks of 0.285 W/m$^2$ in more recent assessments, all remain consistently and significantly below ICNIRP reference limits.

**Fig. 1:**
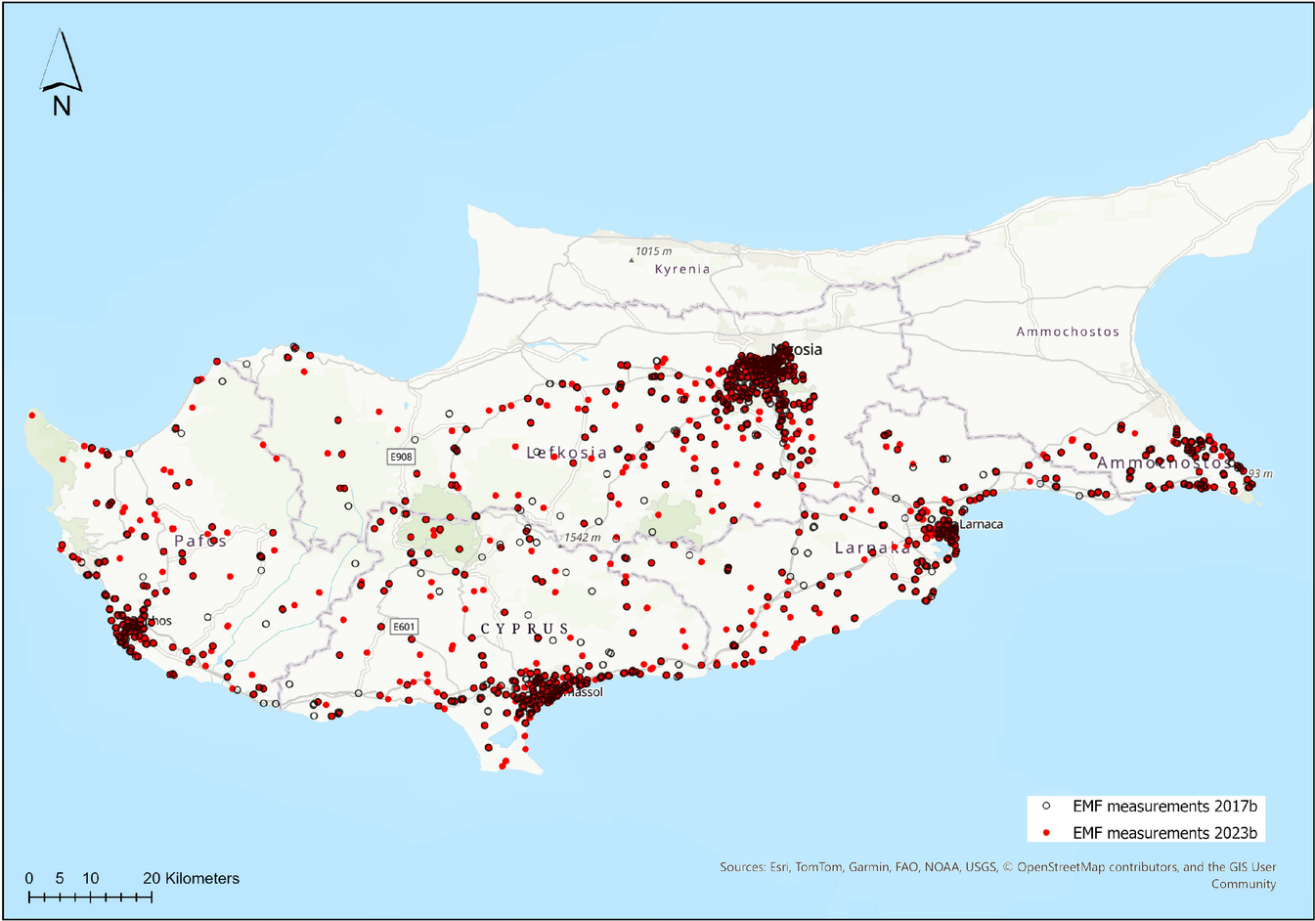
Spatial distribution of EMF monitoring locations across Cyprus, comparing the earliest recorded dataset (2017b; grey points) with the most recent dataset (2023b; red points)

**Table 2:** Frequency bands monitored by the Cyprus national RF-EMF measurement program. Measurements were performed as periodic in-situ spot assessments at fixed locations around operational mobile telephony base stations

Code	Zone description	Frequency range
FM	Radio broadcasting zone	87.5–108 MHz
VHF	Very high frequency (excluding FM)	30–87.5 MHz, 108–300 MHz
UHF-TV	Terrestrial digital television zone	470–790 MHz
700	Mobile telephony zone	694–790 MHz
800	Mobile telephony zone	791–821 MHz
900	Mobile telephony zone	925–959.8 MHz
1800	Mobile telephony zone	1805.2–1880 MHz
2100	Mobile telephony zone	2110–2170 MHz
2600	Mobile telephony zone	2620–2690 MHz
3600	Mobile telephony zone	3400–3800 MHz
*Other frequencies*	Frequency ranges within the spectrum of 30 MHz–6 GHz not covered above	-

## Materials and methods

### Dataset description

The data set used in this study originates from publicly accessible sources provided by data.europa.eu, the official portal for open European data (Publications Office of the European Union, 2025), and is also available through the Cyprus National Open Data Portal, a platform that hosts various data sets specific to Cyprus (Ministry of Communications and Works, Cyprus, 2025). Electromagnetic field measurements were performed using specialized monitoring devices (technical specifications and equipment details are available at the official website of the Department of Electronic Communications (Department of Electronic Communications, 2024) installed at all existing mobile telephony base stations (operated by Cyta and Epic) (Fig. 1). The data collection followed a standardized protocol (European Conference of Postal and Telecommunications Administrations (CEPT), 2002) to ensure accuracy, comparability, and compliance with international exposure limits. This protocol is employed by the Department of Electronic Communications within the Ministry of Research, Innovation, and Digital Policy of Cyprus (see Table 2), which operates the national monitoring network. A key strength of this dataset is the methodological consistency: measurements are conducted at fixed monitoring positions at standardized distances from the emitting sources. This spatial permanence is critical for longitudinal reliability, as it ensures that the temporal trends observed over the 7-year period are not confounded by changes in the measurement environment or positioning. All monitoring activities follow the national regulatory framework, which aligns with European standards for continuous environmental RF-EMF assessment.

**Table 3:** Total number of measurement instances corresponding to each frequency band for the period 2017–2023

Year	FM	VHF	UHF-TV	700 MHz	800 MHz	900 MHz	1800 MHz	2100 MHz	2600 MHz	3600 MHz
2017	1551	1080	1718	–	1238	2374	1776	2142	981	–
2018	2785	2785	2785	–	2785	2785	2785	2785	2785	–
2019a	2754	2754	2754	–	2754	2754	2754	2754	2754	–
2019b	2827	2827	2827	–	2827	2827	2827	2827	2827	–
2020a	1741	1481	1959	–	2423	2611	2324	2480	1807	–
2020b	2159	1983	2297	–	2758	2867	2776	2832	2417	–
2021a	2059	1982	2264	694	2691	2746	2712	2743	2382	548
2021b	3957	3957	3957	3957	3957	3957	3957	3957	3957	3957
2022a	2848	2798	3032	3063	3529	3575	3550	3546	3183	2877
2022b	2791	2642	3008	2975	3591	3620	3545	3540	3029	2687
2023a	2847	2825	3019	3036	3460	3488	3475	3472	3131	2887
2023b	1162	545	1498	2175	2823	2879	2789	2695	1696	1210

Under Cyprus’ national RF-EMF measurement program, periodic in-situ measurements are conducted at multiple fixed locations strategically distributed across urban, suburban, and rural regions to represent diverse exposure environments. These locations are selected based on population density, proximity to radio transmitters and base stations, and land-use characteristics. All measurement equipment, including spectrum analyzers, broadband field meters, and isotropic probes, is periodically calibrated in accredited laboratories. Instrument sensitivity, frequency range, and dynamic range are verified before deployment to guarantee measurement reliability. Measurements are typically performed at a standard height (approximately 1.5–1.7 m above ground level) to reflect typical human exposure conditions. The surrounding environment is kept clear of large metallic objects or reflective surfaces that could distort readings. Environmental parameters such as temperature and humidity are also recorded. Electric field strengths are measured across multiple frequency bands, including FM, VHF, UHF-TV, and mobile communication bands. The monitoring system scans a wide range of frequencies, from low radio bands to several gigahertz. Each frequency band is sampled with an appropriate resolution bandwidth to capture both narrowband and broadband sources of electromagnetic radiation. To capture temporal variations (daily, weekly, and seasonal), the system performs long-term continuous measurements. Results are time-averaged over standardized intervals (e.g., 6 min, hourly, or daily) following ICNIRP and European Commission recommendations. Shorter sampling intervals are used to identify transient peaks or bursts in field strength. All data undergo rigorous quality control to eliminate noise, instrument errors, and outliers. Duplicate measurements and cross-calibration checks are conducted periodically to ensure data consistency and accuracy. Validated data are stored in a central database together with metadata such as site coordinates, measurement time, and instrument configuration. Periodic reports are published summarizing long-term exposure trends, compliance with ICNIRP limits, and variations among different frequency bands (Ministry of Communications and Works, Cyprus, 2025).

Furthermore, the procedure complies with Recommendation CEPT/ECC/REC/(02)04, entitled *Measuring Non-Ionizing Radiation (9 kHz-300 GHz)*. The intensity of the electric field was recorded across the three signal propagation axes (x, y, z). To ensure assessment of the worst-case scenario, the highest recorded electric field intensity values were considered for each emission, instead of the 6-minute average recommended by CEPT/ECC/REC/(02)04. At every measurement location, contributions from all sources of electromagnetic radiation were accounted for. While the use of maximum recorded values enables conservative, worst-case exposure assessment, it may overestimate typical ambient exposure compared to standardized time-averaged metrics. Consequently, direct quantitative comparisons with studies or compliance assessments based on 6-minute averaging should be made with caution.

All frequency zones shown in Table 2 are the official measurement categories of the Cyprus national RF-EMF measurement program and are assessed using frequency-selective spectrum analysis during each periodic visit.

Table 3 summarizes the number of valid measurement instances collected for each frequency band across the study period 2017–2023b. Numbers represent the total count of periodic in-situ measurement instances per band (one per location visit). These are not continuous recordings from fixed sensors. A substantial increase in monitoring coverage is observed from 2021 onward, coinciding with the expansion of Cyprus’s EMF monitoring network and the introduction of new mobile communication technologies such as 3600 MHz bands. The dataset demonstrates strong temporal growth in monitoring density, ensuring robust representation of exposure conditions across both traditional broadcast and modern cellular bands.

### Statistical analysis

In our statistical analysis, the Kruskal-Wallis test was employed to compare the distributions of multiple independent groups and assess whether statistically significant differences existed among them. This nonparametric test was selected because it does not assume normality of the data and is suitable for comparing median values across groups when the underlying distributions may differ in shape or variance. Also, to evaluate monotonic temporal trends in RF-EMF exposure across the study period, the non-parametric Mann-Kendall test was applied to the mean electric field strength values for each frequency band. When a statistically significant result was obtained (*p* < 0.05), it indicated that at least one group’s distribution differed from the others, warranting further post-hoc analysis to determine the specific sources of variation.

### Geospatial mapping and processing pipeline

Geospatial analyses were conducted to derive spatially resolved exposure surfaces and visualize hotspot patterns. All geospatial operations were performed in WGS84 (EPSG:4326). A regular analysis grid was used to summarize station-based measurements to spatial cells and enable map-based comparisons across bands and semesters.

EMF exposure mapping is vital for regulatory compliance and public health, yet unified methodologies remain scarce (Kiouvrekis & Panagiotakopoulos, 2025). To address the challenges of large-scale urban monitoring, one method to address is the Voronoi diagrams (also known as Thiessen polygons) for spatial partitioning. This approach, as recently validated by Arribas et al. (2025), assigns the measured RF-EMF value of a specific station to its surrounding polygonal area. By increasing the density of “seed” points, the model achieves mathematical stabilization, allowing for a clear, discrete visualization of the electromagnetic landscape.

In our research, in order to visualize continuous exposure fields, we applied ordinary kriging with an empirical semivariogram (spherical model, fitted range and sill optimized via leave-one-out cross-validation). Kriging was chosen over IDW to account for spatial autocorrelation; cross-validated mean-square error (MSE) and mean bias were reported for each band/semester. Ordinary kriging was preferred over inverse distance weighting (IDW) due to its ability to model spatial autocorrelation. Cross-validation results indicated superior performance of kriging, with a mean MSE of approximately 2.5 V/m, whereas IDW produced substantially higher errors ($\sim$ 4.5 V/m). Where station density was insufficient, maps were restricted to Voronoi/Thiessen polygons to avoid spurious interpolation.

#### Software

Analyses were performed in Python 3.11 (geopandas, pykrige, numpy, scipy, matplotlib) and QGIS 3.x. Scripts to reproduce the figures are provided (see Data and Code Availability).

### Limitations

While this study establishes a robust baseline for RF-EMF levels in Cyprus, it is limited by its focus on outdoor ambient intensity relative to ICNIRP limits. From a public health standpoint, ambient levels are only a proxy for actual human exposure. A full epidemiological translation would require the conversion of electric field strength into SAR or personal dose-response metrics, which was beyond the scope of this geospatial analysis. Additionally, this study does not account for indoor exposure or the specific pulsing characteristics of 5 G signals.

The presented results are based on in-situ measurements conducted at predefined fixed locations in three distance zones from the base stations, rather than continuous monitoring by fixed stations. While the same general measurement protocol and calibrated equipment were used across campaigns, it was not possible to fully control all parameters for every measurement, including equipment type, measurement uncertainty, specific setup settings (e.g., measurement range, RBW), and the exact time of day or weekday when measurements were taken. These factors may influence the distribution of peak and median values and limit the strict comparability of results across years and frequency bands. Consequently, the analysis should be interpreted as reflecting long-term statistical trends rather than precise absolute values under strictly controlled conditions. Nevertheless, despite this limitation, the use of non-parametric statistics (median-based and rank-based methods) reduces sensitivity to measurement variability and ensures robustness of the identified temporal trends.

Additional limitations specific to the Cyprus national RF-EMF measurement program and the analytical approach must be acknowledged. First, temporal bias from network densification may arise because the number of measurement locations increased substantially after 2020/2021 with the addition of new base stations and the introduction of the 700 MHz and 3600 MHz 5 G bands. Although all measurements were performed at the same fixed locations, the expanding sample may introduce a mild upward bias in national aggregates; however, this potential effect is mitigated through site-specific trend analyses using the Mann-Kendall test and the use of median-focused statistics. Second, artefacts from geostatistical interpolation can appear in areas of low monitoring-location density, particularly in rural Cyprus, where ordinary kriging or Voronoi/Thiessen polygons may produce smoothing artefacts or edge effects. To minimize this, maps were restricted to Voronoi polygons where station density was insufficient for each band and semester. Third, the use of maximum recorded values follows the national protocol and CEPT/ECC/REC/(02)04, whereby the highest electric field intensity across the three axes (x, y, z) is retained as a worst-case scenario. This conservative approach provides an upper-bound exposure estimate but precludes direct numerical comparison with studies that employ 6-minute time-averaged metrics.

Outlier values were also observed across the dataset. Outliers (occasionally reaching or exceeding 25–60 V/m) appeared in every frequency band and semester. These represent genuine worst-case peak readings captured under the protocol (for example, line-of-sight conditions or momentary high-traffic loads near antennas) and were deliberately retained rather than removed. Non-parametric tests (Kruskal-Wallis and Mann-Kendall) are inherently robust to outliers, so all conclusions are based on medians and monotonic trends. In the mobile communication bands (800, 1800, 2100, and 2600 MHz), the gradual increase in both frequency and magnitude of outliers after 2020 is attributable to network densification and rising data demand rather than any regulatory non-compliance. In the broadcast bands (FM and VHF), outliers were rarer and of lower magnitude, consistent with the mature and stable nature of these services. All values, including outliers, remained far below the corresponding ICNIRP reference limits.

**Fig. 2:**
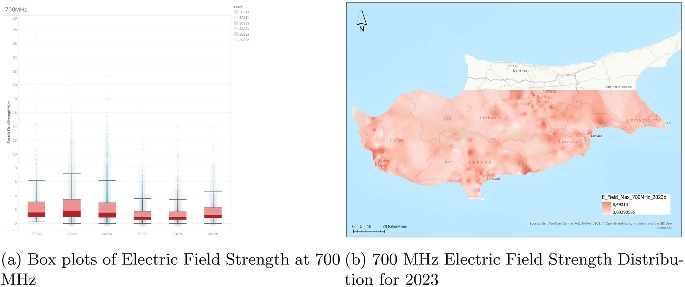
Dual representation of 700 MHz electric field strength data (2023)

Acknowledging these constraints, our findings should be viewed as a foundational environmental assessment that provides the necessary data for future, dedicated epidemiological studies to evaluate long-term, non-thermal health implications in the Cypriot population.

## Results

### Analysis of electric field strength at 700 MHz

The chart (Fig. 2) presents electric field strength measurements at 700 MHz across several half-year periods from the first semester of 2021 (2021a) through the second semester of 2023 (2023b). The vertical axis represents the electric field strength in volts per meter (V/m), while the horizontal axis categorizes the data into six time periods, each corresponding to a different half-year. Each boxplot summarizes the distribution of measured values for its respective period, with the color-coded legend helping to distinguish between the years and their halves.

From the visual analysis, it is evident that the median electric field strength has remained fairly stable across the examined periods, consistently registering below 2 V/m. This indicates a relatively constant average level of exposure over the years. The interquartile range (IQR), which represents the middle 50% of the data, also exhibits similar widths across all time intervals. Notably, there is a subtle compression of the boxes as we move into the 2023 period.

Outliers are present in all years, with the most extreme values observed in the first semester of 2021, reaching up to approximately 30 V/m. However, as time progresses, there is a visible decline in the number and magnitude of these outliers, particularly from 2022 onwards. This decreasing trend in outliers could point to fewer instances of exceptionally high field strengths. Additionally, the whiskers, which represent the range of values excluding outliers, show a general tendency to shorten over time, indicating that the upper bounds of typical measurements have decreased slightly.

While the central tendency of electric field strength remains steady, the data suggests a mild decline in both the frequency and severity of higher exposure levels over the 3-year period. This could potentially be attributed to improvements in infrastructure, more uniform distribution of signal sources, or evolving regulatory practices affecting emission limits.

**Fig. 3:**
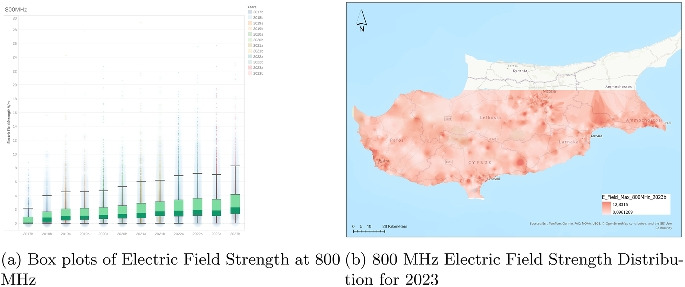
Dual representation of 800 MHz electric field strength data (2023)

### Analysis of electric field strength at 800 MHz

The box plots in Fig. 3 illustrate the electric field strength at 800 MHz over a broad time span from the second half of 2017 (2017b) to the second half of 2023 (2023b). Each box plot corresponds to a half-year interval and provides a statistical summary of the distribution of field strength values measured in volts per meter (V/m). The data shows a consistent upward trend in both the median values and the interquartile ranges (IQRs) over the observed years. In the earlier years, particularly from 2017b to around 2019a, the medians remain relatively low, generally under 2 V/m, with relatively tight interquartile ranges. However, as the timeline progresses, the boxes become slightly taller, indicating a gradual increase in variability and central tendency.

Throughout the period, the presence of outliers is notable. These outliers, which represent particularly high measurements, grow more pronounced in later years, especially after 2020. Some values reach above 30 V/m, suggesting instances of strong localized emissions. The whiskers of the boxplots also lengthen over time, supporting the observation that the typical range of exposure is expanding. This pattern might reflect increased deployment or usage intensity of 800 MHz frequencies, possibly due to expanded mobile network infrastructure or changes in measurement density.

Although the increase in median values and variability is gradual rather than abrupt, the trend is clear: there is a general rise in both the average and spread of electric field strengths at 800 MHz over the 6-year period. These findings may indicate evolving network demands, a wider signal distribution, or updated measurement protocols. The increase in upper outlier values also raises potential considerations for monitoring localized exposure peaks, especially in densely populated or high-usage areas.

**Fig. 4:**
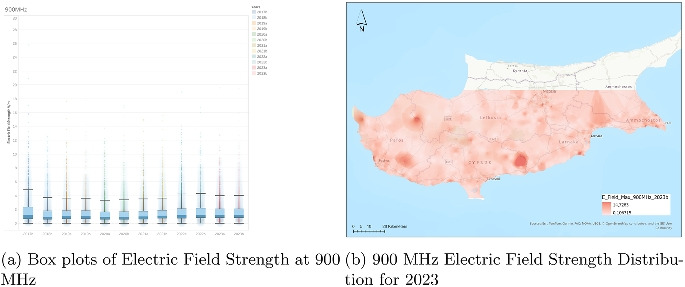
Dual representation of 900 MHz electric field strength data (2023)

### Analysis of electric field strength at 900 MHz

The electric field strength distribution at 900 MHz (Fig. 4) is depicted across a time series from the second half of 2017 (2017b) to the second half of 2023 (2023b), with each boxplot representing half-year intervals. Measured in volts per meter (V/m), the values reveal a fairly stable trend throughout the examined period. The median electric field strength remains consistently low, generally around or slightly above 1 V/m, with only minimal fluctuation between the time intervals. This suggests that the average exposure levels have not changed significantly over the 6-year span.

The interquartile ranges (IQRs), representing the spread of the central 50% of data, are also relatively constant, reflecting a consistent distribution of typical exposure levels. Although there are visible outliers present in every period, some reaching values greater than 20 V/m, these instances are infrequent and appear to be isolated cases rather than indicative of broader trends. The whiskers, which mark the range excluding outliers, remain similar in length across all periods, further emphasizing the stability of the measured values over time.

The 900 MHz chart does not show a clear trend of increasing central tendency or variability. Instead, it portrays a plateau in both the average and the distribution of electric field strengths, suggesting that the deployment or intensity of 900 MHz transmissions may have reached a level of maturity earlier than the other bands. The consistency of the data implies effective regulatory or infrastructural control over emission levels, with no major shifts in signal behavior during the time span.

**Fig. 5:**
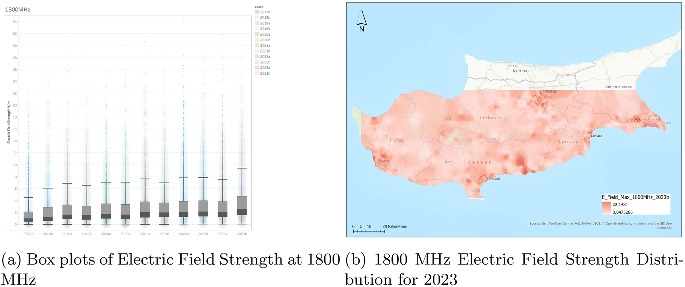
Dual representation of 1800 MHz electric field strength data (2023)

### Analysis of electric field strength at 1800 MHz

The visualization (Fig. 5) for the 1800 MHz band captures electric field strength data from the second half of 2017 (2017b) through the second half of 2023 (2023b), with each boxplot representing a half-year period. The measured values, expressed in volts per meter (V/m), display a relatively stable pattern in terms of central tendency. Throughout the timeline, the medians consistently remain close to or slightly above 2 V/m, with only modest fluctuations from period to period. This suggests a steady average exposure level throughout the years.

However, compared to lower-frequency bands, the interquartile ranges (IQRs) in the 1800 MHz band appear somewhat wider, implying a broader distribution of typical field strengths. This widening becomes more noticeable in later years, especially from 2020 onwards. Furthermore, outliers are persistently present in all intervals, with numerous values exceeding 30 V/m and some approaching or exceeding 35 V/m. These outliers tend to increase slightly in both frequency and magnitude over time, pointing toward more frequent occurrences of elevated field strength values, likely due to increasing network load or intensified infrastructure use in this frequency band.

The upper whiskers of the boxplots gradually extend over the years, reinforcing the notion of expanding exposure variability. Despite the general stability of median values, the upward movement in maximum non-outlier values suggests that peak exposure levels within standard operating ranges have incrementally increased. This could be attributed to the continued rollout of high-density cellular infrastructure utilizing the 1800 MHz band or to changes in traffic patterns and data consumption behaviors.

In summary, while the average exposure in the 1800 MHz band has remained largely unchanged, the increasing spread and intensity of higher readings indicate a slow but steady intensification of usage. This pattern may warrant further investigation into potential localized effects, particularly in areas with heavy deployment or frequent high-demand usage.

**Fig. 6:**
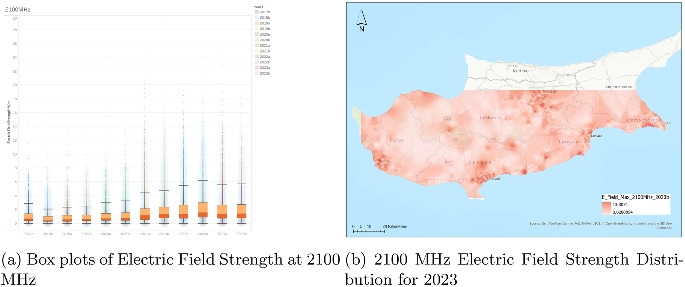
Dual representation of 2100 MHz electric field strength data (2023)

### Analysis of electric field strength at 2100 MHz

The electric field strength measurements at 2100 MHz ((Fig. 6)), spanning from the second half of 2017 (2017b) to the second half of 2023 (2023b), reveal a relatively consistent distribution of values with subtle upward trends. Each boxplot represents a half-year and shows the central tendency, variability, and outlier presence of field strength data expressed in volts per meter (V/m). The median values remain steadily low, typically around 1.5 to 2 V/m, across all time periods, indicating that the average exposure level has not shifted significantly over time.

Despite stable medians, the interquartile ranges (IQRs) show a gradual expansion over the years. This broadening suggests that while the typical field strength has remained similar, there has been a slight increase in variability among measurements. Outliers, although present throughout the time series, appear more frequently and with higher magnitudes in the more recent years, especially after 2020. Some measurements reach up to or beyond 25 V/m, though these remain isolated and infrequent.

The upper whiskers of the boxplots also tend to lengthen over time, particularly from 2020 onward, indicating that nonoutlier high values have been climbing slightly. This may reflect a growing utilization of the 2100 MHz band, likely due to expanded mobile network capacity, especially in urban and high-demand regions. The consistent yet slightly intensifying data distribution suggests the gradual evolution of network load and infrastructure rather than abrupt changes in emission patterns.

In conclusion, the 2100 MHz electric field strength data reveals a scenario of stable average exposure accompanied by a mild increase in variability and high-end values over the observed 6-year period. While no dramatic shifts are evident, the pattern supports a narrative of steady infrastructure development and possibly higher user demand in this frequency range.

**Fig. 7:**
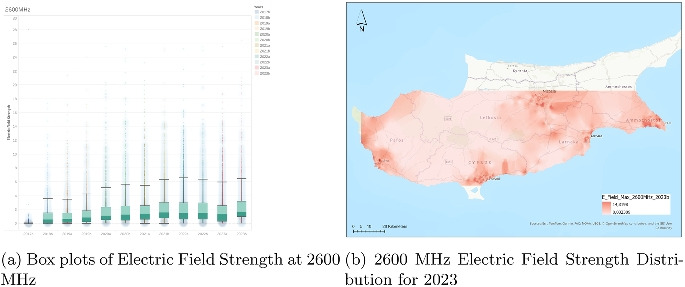
Dual representation of 2600 MHz electric field strength data (2023)

### Analysis of electric field strength at 2600 MHz

The electric field strength data for the 2600 MHz band (Fig. 7), spanning from the second half of 2017 (2017b) to the second half of 2023 (2023b), demonstrate a gradual increase in both the variability and upper-end intensity of measured values. The boxplots represent half-year intervals and show the distribution of field strength in volts per meter (V/m), capturing the evolution of exposure over time. The median values are relatively steady, remaining consistently between 1.5 and 2.5 V/m across the years, indicating a stable average exposure level.

Despite the stable central tendency, there is a noticeable widening of the interquartile range (IQR) starting from around 2020. This indicates an increase in variability within the typical range of measurements. The upper whiskers, which represent the boundary of non-outlier data, also expand over time, showing that the range of commonly occurring higher values has grown. The presence of outliers is a consistent feature of the data set, and an exceptionally high value above 60 V/m appears in 2017b, although it stands out as a rare anomaly. In the later years, particularly from 2021 onward, outliers frequently reach above 20 V/m, with several approaching or exceeding 25 V/m.

These patterns suggest that the 2600 MHz band has seen increased utilization and perhaps more intensive deployment of infrastructure over time. The rise in high-end values may reflect a higher density of transmissions or increased demand on the network, especially in urban or high-capacity areas. The fact that this expansion has occurred while median values have remained largely stable implies that the general environment has not drastically changed for most users, though specific locations may experience higher localized exposure.

In conclusion, the 2600 MHz data reveal a trend of increasing variability and higher peak values over time, while maintaining a relatively consistent average exposure. This dual behavior likely reflects a maturing yet expanding deployment, balancing stable widespread usage with growing intensity in select hotspots.

**Fig. 8:**
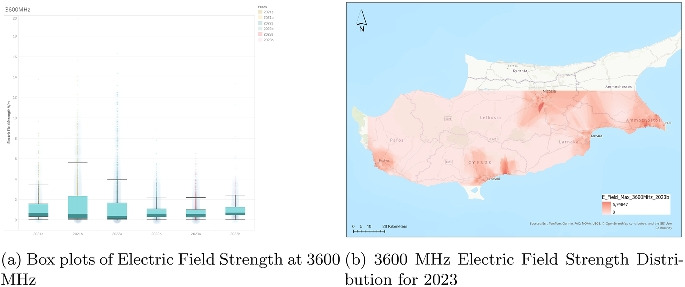
Dual representation of 3600 MHz electric field strength data (2023)

### Analysis of electric field strength at 3600 MHz

The chart for the 3600 MHz frequency band (Fig. 8) provides electric field strength data from the first half of 2021 (2021a) to the second half of 2023 (2023b). As one of the newer and higher-frequency bands, likely associated with the 5 G rollout, the data span only 3 years, but still reveal several notable patterns. Each half-year is represented as a boxplot showing field strength in volts per meter (V/m), capturing both the central tendency and the spread of the measurements.

The median electric field strength values are low across all time intervals, generally below 2 V/m, indicating a modest average exposure level. The interquartile ranges are also narrow, suggesting relatively low variability among the central 50% of data. However, the presence of outliers is notable in the earlier periods, particularly 2021b, where a few extreme values exceed 25 V/m, and one even surpasses 30 V/m. In subsequent years, the number and magnitude of outliers appear to decline slightly, potentially indicating more controlled or optimized signal deployment as the network matured.

Although the overall shape of the boxplots remains consistent throughout the periods, there is a subtle contraction of the whiskers over time. This suggests that the general range of field strengths experienced a mild narrowing, possibly reflecting stabilization of infrastructure and emission patterns. The slight decline in maximum values may also point to fine-tuning of power levels and improved spatial distribution of transmitters as the deployment progressed.

In summary, the 3600 MHz band shows a pattern of initially variable but quickly stabilizing electric field strengths. The use of the band appears to have increased in 2021 with relatively high variability, followed by more consistent exposure levels in later periods. This aligns well with early-phase infrastructure deployment followed by operational refinement in newer network layers such as 5 G.

**Fig. 9:**
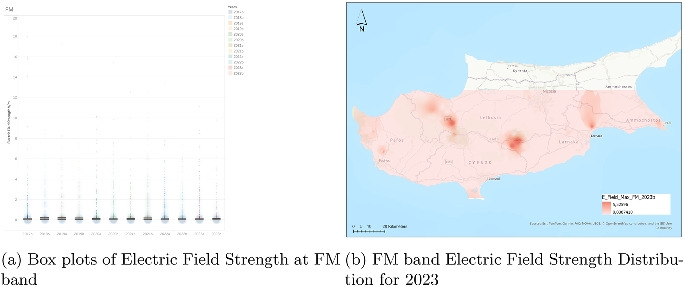
Dual representation of fm band electric field strength data (2023)

### Analysis of electric field strength in the FM Band

The FM band electric field strength data (Fig. 9) spans from the second half of 2017 (2017b) through to the second half of 2023 (2023b), offering insight into the long-term behavior of a mature and well-established frequency range. Each half-year interval is represented by a boxplot that captures the distribution of electric field strength values in volts per meter (V/m). Across this entire period, the data exhibits remarkable consistency in both central tendency and variability.

The median values remain low, typically under 1 V/m, and show almost no deviation over the years. This consistent median reflects a stable average exposure level, characteristic of a frequency band that has seen little technological or infrastructural change during this time. The interquartile ranges (IQRs) are narrow, indicating a tightly clustered dataset with limited variability within the central 50% of values. These attributes confirm that the FM band maintains a predictable and regulated presence in the electromagnetic environment.

Outliers, while present in every interval, do not dominate the distribution and are generally confined below 20 V/m. Their presence remains fairly uniform over time, suggesting that these rare spikes are likely tied to specific, localized conditions rather than systemic changes in FM broadcasting. The whiskers, representing the spread of non-outlier values, also stay relatively short and constant, further reinforcing the conclusion of long-term stability.

In conclusion, the FM band stands out for its low and stable exposure levels over a multi-year period. Unlike higher-frequency mobile communication bands, FM radio exhibits no signs of intensifying or expanding signal strength. This behavior aligns with the static nature of FM infrastructure and its mature role in the overall electromagnetic spectrum.

**Fig. 10:**
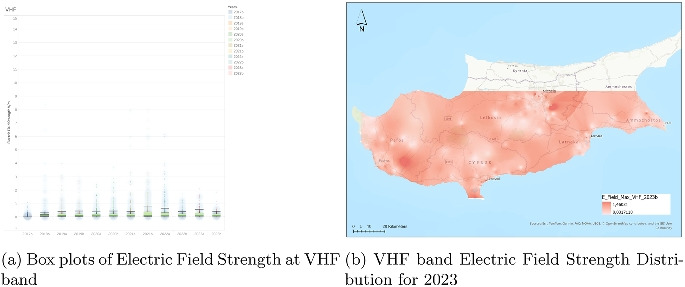
Dual representation of VHF band electric field strength data (2023)

### Analysis of electric field strength in the VHF band

The box plots (Fig. 10) for the VHF band illustrate electric field strength measurements from the second half of 2017 (2017b) through the second half of 2023 (2023b), covering a broad time frame and offering insight into long-term patterns for this traditionally stable frequency range. Each half-year period is depicted as a boxplot showing the distribution of field strength in volts per meter (V/m). Throughout the duration, the median values remain consistently low, generally below 1 V/m, demonstrating the characteristically low exposure levels associated with VHF transmissions.

The interquartile ranges are narrow throughout the time series, indicating low variability in the typical measurement values. There is little evidence of any significant trend, either upward or downward, in central tendency or spread, suggesting that the use of VHF frequencies has remained highly stable. Outliers are present but sparse, and in most periods, they do not exceed 8 V/m, with a few exceptions scattered sporadically. These outliers are isolated in nature and do not indicate any systemic increase in exposure levels.

The whiskers, which represent the limits of the data that exclude outliers, are short and consistent throughout all periods. This further confirms that the vast majority of field strength readings remain within a narrow and unchanging band, reinforcing the static nature of VHF infrastructure, which typically includes television broadcasting and other low-bandwidth services.

**Fig. 11:**
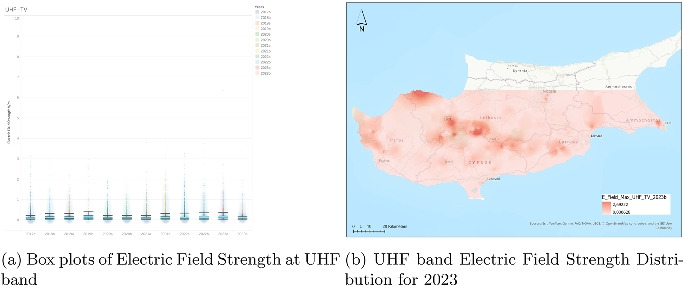
Dual representation of UHF band electric field strength data (2023)

In conclusion, the VHF band exhibits one of the most stable and predictable electric field strength profiles over the observed years. With low medians, minimal variability, and infrequent outliers, the data reflects the long-standing maturity and conservative usage of this part of the spectrum. No signs of intensification or systemic change are present, marking VHF as a passive background element in the broader electromagnetic landscape.

### Analysis of electric field strength in the UHF-TV band

The electric field strength data for the UHF-TV band (Fig. 11) covers the period from the second half of 2017 (2017b) through to the second half of 2023 (2023b). Each half-year interval is represented as a boxplot, displaying the distribution of field strength values in volts per meter (V/m). Throughout this span, the dataset reflects a stable and consistently low level of exposure, with no substantial shifts in central tendency or distribution. Median values remain well below 1 V/m, reinforcing the passive and non-evolving nature of UHF television broadcast infrastructure over time.

The interquartile ranges (IQRs) are narrow and show minimal fluctuation, suggesting that the majority of measured values are tightly clustered around the median. This stability across multiple years points to a highly regulated and mature use of this frequency band. The whiskers, representing the extent of nonoutlier data, are short and uniform across nearly all periods, indicating that typical exposure values do not vary significantly. While outliers do occur sporadically, they are sparse and largely limited to isolated values below 5 V/m, with only a few readings exceeding this level, notably in the 2023 intervals.

Overall, the data for UHF-TV indicates minimal variability and extremely low average exposure, reflecting the static nature of over-the-air television broadcasting in the UHF range. This pattern is consistent with the long-standing deployment of television infrastructure and the lack of substantial evolution in how this band is used. There is no indication of increasing intensity or variability, confirming the UHF-TV band’s role as a reliably stable component of the electromagnetic environment.

### Comparative summary across frequency bands

Across the broad range of frequency bands analyzed, from traditional broadcast bands like FM, VHF, and UHF-TV to modern mobile communication bands spanning 700 to 3600 MHz, distinct trends in electric field strength and variability emerge. The FM, VHF, and UHF-TV bands exhibit exceptionally low median electric field strengths, generally below 1 V/m, with minimal variability over time. These bands show little to no change throughout the years, reflecting their mature and static usage in broadcasting applications.

In contrast, mobile communication bands display greater variation in both central tendency and spread. The 700 MHz, 800 MHz, and 900 MHz bands have moderate median values (between 1.5 and 2.5 V/m), with the 800 MHz band showing a gradual increase in both variability and exposure in the upper range. The 1800 MHz and 2100 MHz bands show slightly higher medians and greater interquartile ranges, indicating more active usage and potentially denser deployment environments. The 2600 MHz band marks a noticeable uptick in both field strength variability and outlier frequency, while still maintaining stable medians. Finally, the 3600 MHz band, representing one of the newest bands associated with 5 G, initially exhibits high variability and sporadic high outliers, but shows a stabilization trend in recent years, suggesting early deployment effects that transition into mature usage patterns.

**Fig. 12:**
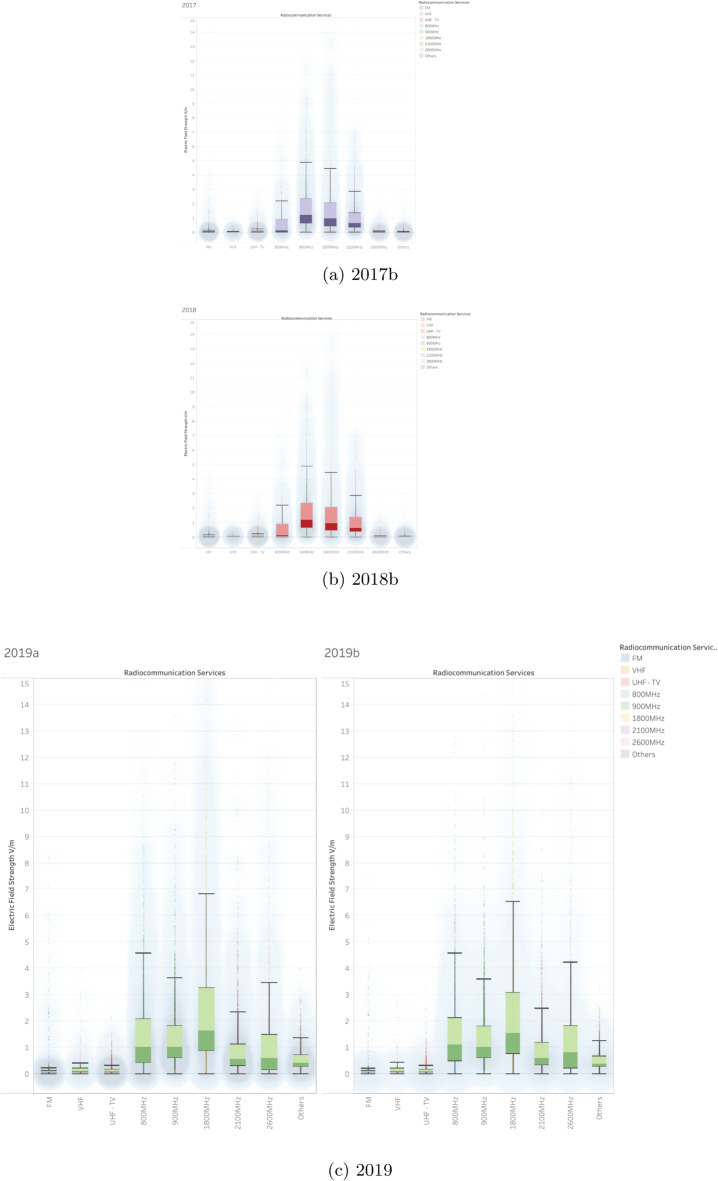
Electric field strength distributions across years 2017–2019

### General trends and evolution over the years

Across the observation period, distinct patterns emerge in the evolution of electric field strength for different frequency bands. The 700 MHz and 3600 MHz bands, which have data available starting from 2021, initially display higher variability, particularly during their first recorded year, but gradually stabilize by 2023. This stabilization likely corresponds to the controlled rollout and integration of these bands into existing network infrastructure. In contrast, other frequency bands such as 800 MHz, 900 MHz, 1800 MHz, 2100 MHz, 2600 MHz, FM, VHF, and UHF-TV, which have been monitored since 2017, show a more consistent upward trend, particularly around the year 2021. This timing aligns with notable expansions in mobile network infrastructure, including preparations for 5 G deployment.

Outliers in electric field measurements are most prominent in higher-use cellular bands such as 2600 MHz, 1800 MHz, 2100 MHz, 800 MHz, and 900 MHz. In these cases, peak values occasionally range between 25 and 60 V/m, significantly exceeding typical values but remaining relatively rare in occurrence. These anomalies may reflect specific high-density urban zones or locations near transmission sources. In contrast, broadcast-oriented frequencies such as FM, VHF, and UHF-TV exhibit far fewer and less intense outliers, pointing to their long-established, low-variability transmission patterns.

**Fig. 13:**
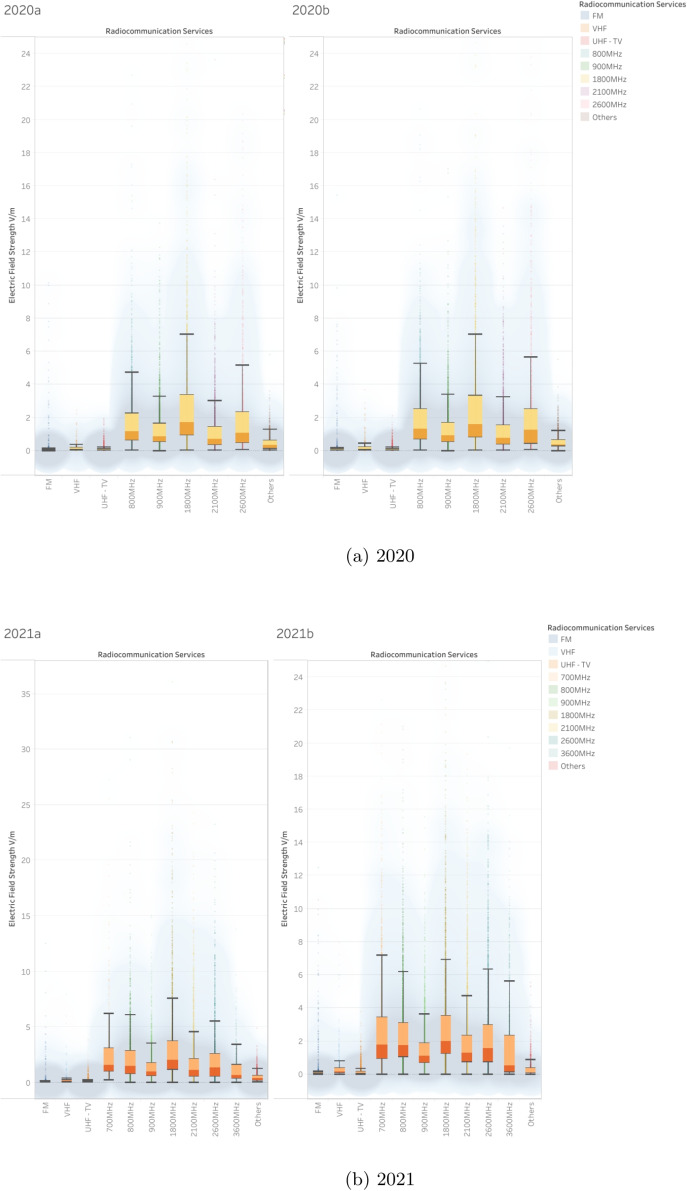
Electric field strength distributions across years 2020–2021

**Fig. 14:**
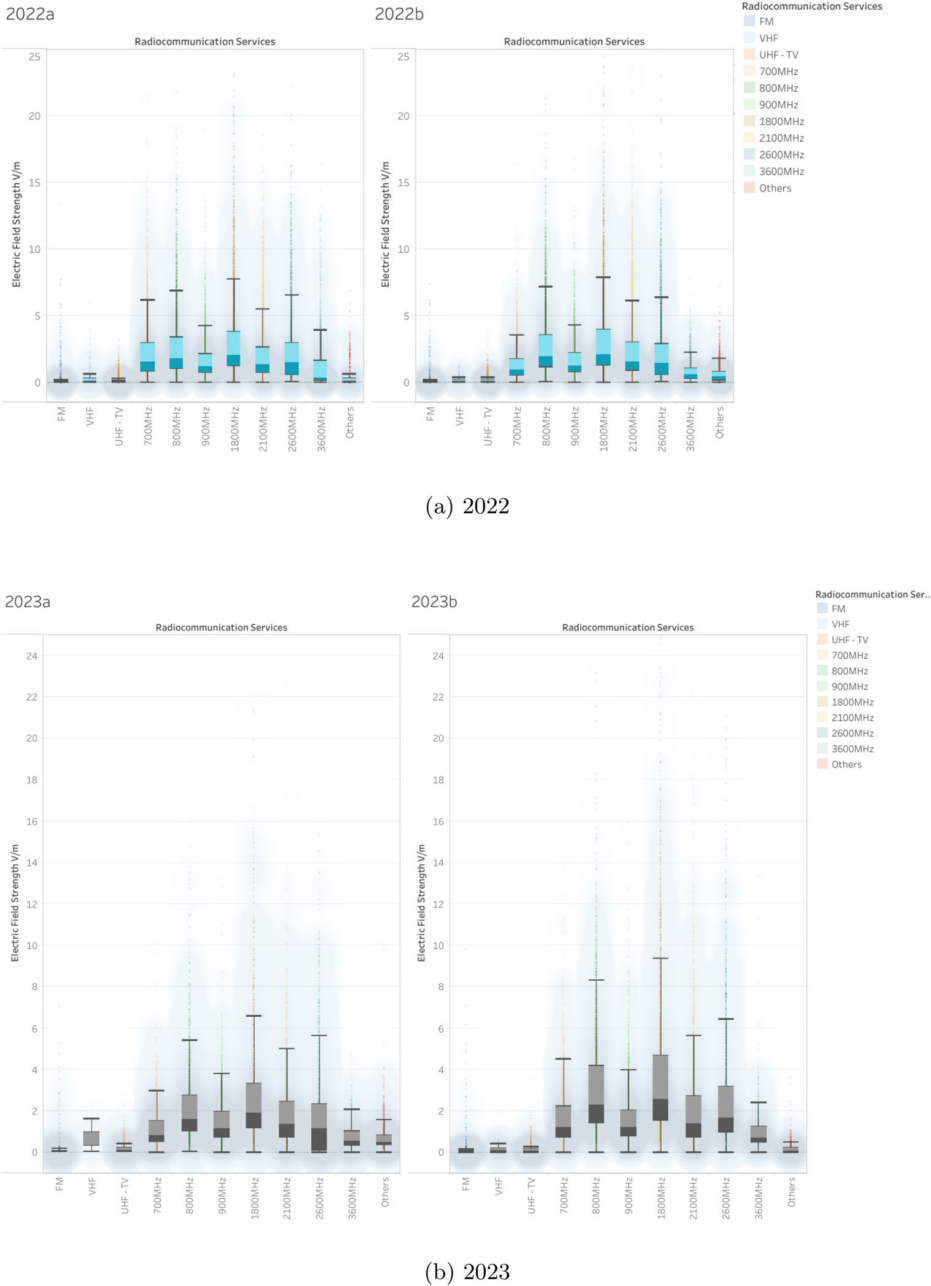
Electric field strength distributions across years 2022–2023

There is a clear distinction between lower and higher cellular frequencies in terms of measured field strength and variability. Frequencies such as 1800 MHz, 2100 MHz, and 2600 MHz tend to register higher median values and broader distributions than lower-frequency counterparts like 700 MHz, 800 MHz, and 900 MHz. This suggests more intense and widespread use of the higher bands in modern mobile network infrastructure. The 3600 MHz band, while still relatively new, shows moderate median values and a controlled spread, indicating its emerging role, particularly in early 5 G deployments. Meanwhile, broadcast bands, including FM, VHF, and UHF-TV, consistently report low electric field strengths, underscoring their static usage and limited exposure footprint.

The data set generally reflects strong compliance with international safety standards for electromagnetic field exposure. Median electric field strength values across all frequency bands remain well below the commonly accepted thresholds, such as those set by ICNIRP (typically between 41 and 61 V/m depending on frequency). Although some peak values, particularly in the higher cellular bands, approach these limits, they do so infrequently and remain within regulatory limits.

Overall, cellular frequency bands ranging from 800 to 2600 MHz exhibit higher variability and a greater occurrence of outliers, which aligns with their intensive usage and continued network expansion. In contrast, broadcast frequencies such as FM, VHF, and UHF-TV remain stable and consistently low in field strength, highlighting their established infrastructure and minimal fluctuation. Newly adopted bands such as 700 MHz and 3600 MHz demonstrate evolving characteristics, likely due to recent technological developments and integration into 5 G services.

### Comprehensive analysis of electric field strength measurements (2017–2023)

#### General observations

Over the course of the 6-year observation period (Figs. 12, 13, and 14), there has been a gradual increase in electric field strengths within the cellular frequency bands, particularly from 2019 through 2023. This trend suggests ongoing infrastructure deployment and increased usage intensity. Conversely, the broadcast bands, FM, VHF, and UHF-TV, have maintained consistently low and stable values throughout the entire monitoring period, reinforcing their predictable and well-regulated nature.

From 2017 to 2018, the dataset primarily features values in the 900 MHz, 1800 MHz, and 2100 MHz bands, which dominate early measurements. A notable rise in electric field strength becomes evident from 2019 to 2020, especially within the 1800 MHz, 2100 MHz, and 2600 MHz bands, reflecting increased deployment and data usage. Between 2021 and 2023, two new frequency bands, 700 MHz and 3600 MHz, enter the dataset, reflecting their introduction as part of new-generation networks. During this period, significant peaks are also recorded in the 1800 MHz and 2100 MHz bands, occasionally reaching 25 to 35 V/m.

Cellular bands such as 800 MHz, 900 MHz, 1800 MHz, 2100 MHz, and 2600 MHz generally display higher median electric field strengths, along with increased variability from year to year. The 700 MHz and 3600 MHz bands, introduced more recently, show a steady increase in field strength as their deployment progresses. In contrast, the FM, VHF, and UHF-TV bands continue to report low, stable exposure levels, reflecting their limited role in evolving communication infrastructure.

The most notable anomalies are observed in the 1800 MHz and 2100 MHz bands, which exhibit wide data spread and occasional spikes in electric field strength. These high-variability measurements likely correspond to areas with high user density or concentrated transmission sources, such as near base stations or urban centers.

Across all bands and measurement periods, median electric field strengths consistently remain well below the ICNIRP safety thresholds of approximately 41 to 61 V/m. Even though certain measurements in cellular bands approach these upper limits, they do so infrequently and without breaching compliance. The overall data profile supports the conclusion that observed exposure levels are in line with international regulatory guidelines, ensuring public safety.

### Hypothesis tests

As part of the statistical analysis, we performed normality tests on the electric field strength data for each frequency band under study. These tests were conducted to evaluate whether the data distributions conformed to a normal distribution, which is a common assumption in many parametric statistical methods. However, the results indicated that none of the frequency bands exhibited a normal distribution. Given this outcome, we proceeded with non-parametric statistical methods, specifically the Kruskal-Wallis test, which does not rely on the assumption of normality and is more appropriate for the analysis of skewed or ordinal data.

#### Kruskal-Wallis test summary

To evaluate whether there were statistically significant differences in electric field strength distributions among the various frequency bands for each half-year period from 2017b to 2023b, the Kruskal-Wallis test was applied. The test was conducted at two significance levels, $\alpha = 0.05$ and $\alpha = 0.01$, across all periods. In every case, the resulting *p*-value was < 0.001, indicating a highly significant result. Consequently, the null hypothesis, which states that there is no statistically significant difference between the distributions, was consistently rejected. These findings imply that at least one frequency band differed significantly from the others in each time period. This consistent outcome over multiple years and significance thresholds strongly suggests systematic variability in exposure levels across different frequency bands.

#### Kruskal-Wallis test: Frequency-Wise Analysis over time

To assess whether there were statistically significant changes in electric field strengths within each frequency band over time, the Kruskal-Wallis test was applied separately to each band. The analysis was conducted using two significance levels, $\alpha = 0.05$ and $\alpha = 0.01$, to ensure robustness of the findings. For all frequency bands, ranging from FM, VHF, and UHF-TV to mobile communication bands such as 700 MHz, 800 MHz, 900 MHz, 1800 MHz, 2100 MHz, 2600 MHz, 3600 MHz, and others, the test consistently returned extremely small *p*-values (e.g., *p* < 0.001 or values approaching machine precision). These results provide strong evidence to reject the null hypothesis in all cases, indicating that for every frequency band analyzed, there were statistically significant differences in the electric field strength measurements across the different years.

**Table 4:** Mann-Kendall trend test results for mean electric field strength across frequency bands (2017–2023b)

Frequency band	Start	End	n	Trend	Significant	*p*-value
1800 MHz	2017	2023b	12	Increasing	Yes	$4.70 \times 10^{-4}$
2100 MHz	2017	2023b	12	Increasing	Yes	$7.79 \times 10^{-4}$
2600 MHz	2017	2023b	12	Increasing	Yes	$1.62 \times 10^{-4}$
3600 MHz	2021a	2023b	6	No trend	No	0.1329
700 MHz	2021a	2023b	6	No trend	No	0.1329
800 MHz	2017	2023b	12	Increasing	Yes	$1.56 \times 10^{-5}$
900 MHz	2017	2023b	12	No trend	No	0.1926
FM	2017	2023b	12	No trend	No	0.9453
UHF-TV	2017	2023b	12	Increasing	Yes	0.01639
VHF	2017	2023b	12	Increasing	Yes	0.001269

**Table 5:** Synthetic summary of Mann-Kendall trend results grouped by service category (2017–2023b)

Bands included	Trend pattern	Overall interpretation
FM, VHF, UHF-TV	Mixed (VHF, UHF increasing; FM stable)	Mostly stable to moderate increase
800, 900, 1800, 2100 MHz	Predominantly increasing (except 900 MHz)	Significant upward trend
2600 MHz	Increasing	Significant upward trend
700, 3600 MHz	No significant trend	Preliminary, short observation period

#### Mann-Kendall trend test

The Mann-Kendall test (Table 4) revealed statistically significant increasing monotonic trends in several cellular communication bands, specifically 800 MHz, 1800 MHz, 2100 MHz, 2600 MHz, as well as in the VHF and UHF-TV broadcasting bands (*p* < 0.05). These upward trends align with the continued expansion and densification of mobile network infrastructure during the study period. In contrast, no significant temporal trends were detected for the FM and 900 MHz bands, indicating stable exposure levels in long-established frequency allocations. Likewise, the recently introduced 700 MHz and 3600 MHz bands did not exhibit significant trends, likely due to the shorter monitoring period available following their deployment in 2021.

### Integrated multiband exposure trends

Across frequency categories, Mann-Kendall analysis indicates a clear divergence between legacy broadcast services and mobile communication bands (see Table 5). Most cellular bands operating since 2017 (800, 1800, 2100, and 2600 MHz) exhibit statistically significant increasing trends, consistent with progressive network densification and traffic growth. In contrast, traditional broadcast services (FM) and certain legacy bands (900 MHz) remain stable, while recently introduced 5 G bands (700 and 3600 MHz) show no statistically significant trends over the shorter observation period. When considered collectively, aggregated multiband exposure demonstrates a significant but gradual upward trend, while remaining well below international guideline limits. This integrative perspective complements the band-resolved analysis and provides a more synthetic representation of national ambient RF-EMF dynamics.

## Conclusions

This temporal study of electromagnetic field (EMF) bands in Cyprus from 2017 to 2023 reveals a distinct divergence in behavior between traditional broadcast bands and contemporary mobile communication frequencies. Broadcast frequencies such as FM, VHF, and UHF-TV demonstrate long-term stability with minimal changes in both median exposure values and variability. These results reflect their static infrastructure, low transmission power, and conservative regulatory constraints.

Several European countries have established dedicated continuous fixed-sensor RF-EMF monitoring networks that provide true 24/7 temporal resolution of environmental exposure levels. These systems offer valuable reference points for interpreting the results of the present study, which is based on periodic in-situ measurements. For example, the Greek National Observatory of Electromagnetic Fields (NOEF) (Greek Atomic Energy Commission (EEAE), 2025) operates a network of approximately 500 fixed monitoring stations equipped with Narda AMB-8057 broadband and selective sensors. Data from this network indicate that median electric field values across mobile communication frequency bands are typically below 0.5 V/m, while contributions from emerging 5 G technologies remain minimal. Similarly, the Serbian EMF RATEL monitoring network (Regulatory Authority for Electronic Communications and Postal Services (RATEL), 2025), consisting of more than 90 fixed stations, has documented moderate increases in exposure levels within the 800, 1800, and 2100 MHz bands following 2020; however, all recorded values remain well below the exposure limits recommended by ICNIRP. Comparable monitoring infrastructures are also implemented in several other European countries, including Romania (approximately 150 stations) (National Authority for Management and Regulation in Communications (ANCOM), 2025) and France (Exem, 2025). The study presented in Jawad et al. (2024), which refers to the French Agence nationale des frequences (ANFR) (Agence Nationale des Fréquences, 2025), describes a national system responsible for monitoring public exposure to electromagnetic fields (EMF). Thousands of in-situ measurements are conducted annually. Their analysis established an empirical day-night interval, showing higher average exposure levels during daytime (08:00–23:00) compared to nighttime (23:00–08:00) for probes located near base stations. Such an analysis is not directly applicable in our case, since our dataset does not include comparable in-situ measurements. Using Principal Component Analysis (PCA), the authors also demonstrated a strong correlation between the exposure levels measured by the probes and the density of nearby cellular base station antennas, which is consistent with the statistical trends observed in our results. Similarly, the study in Karastergios et al. (2020), based on measurements from the Greek National Observatory of Electromagnetic Fields (NOEF) (Greek Atomic Energy Commission (EEAE), 2025), reported that 100% of all measured EMF values were below 10 V/m, remaining well within the national safety limits, which are among the strictest in Europe (set at 60–70% of the EU/ICNIRP reference levels). The vast majority of measurements (over 90%) exhibited annual average values below 1–2 V/m, indicating that typical public exposure is very low across the main frequency bands (900 to 2100 MHz). However, most of the measurements in these studies were not conducted in close proximity to base station antennas, unlike the locations considered in our work. Therefore, the results are not fully comparable. This highlights the need for the development of a universal measurement and evaluation protocol to enable consistent comparisons across different monitoring networks.

In contrast to these autonomous continuous monitoring networks, the RF-EMF monitoring program implemented in Cyprus is based on periodic measurements conducted at fixed locations surrounding each operational base station. Despite these methodological differences, the observed exposure levels are highly consistent with those reported in countries operating continuous monitoring systems. In particular, the median values obtained in the present study (0.5–2.5 V/m), as well as the upper-range measurements, are broadly comparable to those reported by the Greek and Serbian monitoring networks. This consistency indicates that population exposure levels remain several orders of magnitude below the reference limits, even in the context of ongoing network densification and the deployment of 5 G infrastructure. Such cross-national agreement further reinforces the evidence supporting the safety of the current telecommunications infrastructure.

This long-term monitoring study demonstrates clear divergence in the temporal evolution of RF-EMF exposures between traditional broadcasting bands and ex-panding mobile communication frequencies in Cyprus. FM, VHF, and UHF-TV bands exhibited low and stable exposure levels, consistent with the static nature of broadcast services. In contrast, Mann-Kendall trend analysis revealed statistically significant positive monotonic trends in 800 MHz, 1800 MHz, 2100 MHz, and 2600 MHz bands, indicating increasing usage intensity associated with the progressive deployment and densification of 4 G/5 G infrastructure. No significant trends were observed for FM, 900 MHz, 700 MHz, or 3600 MHz bands.

The longitudinal analysis highlights a clear divergence between legacy and modern communication technologies. Broadcast FM remains remarkably stable (*p* = 0.9453), reflecting a mature technological landscape. In contrast, mobile communication bands (800, 1800, 2100, and 2600 MHz) demonstrate significant upward trends (*p* < 0.001), primarily driven by the deployment of 5 G infrastructure following the 2020 spectrum auction and 2021 commercial launch. Although VHF and UHF-TV also showed unexpected increases, all measured levels across the national network remain significantly below ICNIRP safety thresholds.

Across all bands, median exposure values remained far below ICNIRP reference levels throughout the study period, confirming that the technological evolution of communication systems is compatible with current public safety frameworks. Nonetheless, the increasing trend and higher peak values observed in modern cellular bands highlight the importance of maintaining robust surveillance systems capable of capturing localized peaks and future network upgrades. The outcomes support continued transparent communication with the public, spatially-targeted compliance assessments in hotspot locations, and the development of adaptive regulatory strategies aligned with rapidly evolving wireless technologies.

The analysis shows that cellular bands, particularly those used for 4 G and 5 G technologies (including 1800 MHz, 2100 MHz, 2600 MHz, and 3600 MHz), exhibit increased variability and higher peak electric field values over time. This pattern aligns with the intensified use of mobile networks and the progressive densification of infrastructure in urban and high-demand areas. The introduction and gradual deployment of newer bands like 700 MHz and 3600 MHz post-2020 correlate with the launch of new mobile technologies, particularly 5 G, leading to changes in exposure distributions. Also, the findings for 5 G bands reflect early deployment conditions and should not be interpreted as long-term temporal trends.

The post-2021 increase in spatial coverage of monitored locations and the number of periodic measurement campaigns may contribute to enhanced detection of higher local exposure levels. However, the use of standardized protocols and the persistence of increasing trends in band-wise summary statistics indicate that the observed temporal patterns cannot be explained solely by improved spatial coverage.

From an environmental monitoring perspective, the findings support: (i) continuous, spatially resolved surveillance as infrastructures evolve; (ii) publication of transparent, map-based risk communication materials to build public trust; and (iii) targeted audits in hotspot cells where the upper whiskers approach regulatory references, especially during high-traffic periods or special events.

The use of the Kruskal-Wallis test provided robust evidence that EMF exposure levels are statistically dependent on both frequency band and time period. Importantly, while some extreme outliers were recorded, particularly in higher-frequency cellular bands, median values for all bands remained well below international safety thresholds set by the ICNIRP. However, the observed rise in upper-bound exposure levels, especially in the 2600 MHz and 3600 MHz bands, suggests a growing need for proactive, adaptive monitoring policies. Observed trends reflect empirical associations and do not imply causality. Multiple unmodeled factors, including regulatory, technical, and temporal influences, may contribute alongside network densification.

For public health stakeholders, the observed distributions argue for proportionate communication: typical exposures remain low relative to reference levels, yet trend-aware monitoring is warranted to detect local peaks, validate compliance, and inform siting and power-management policies.

In summary, the results indicate that current EMF exposure levels remain within established safety limits, while reflecting the evolving characteristics of modern communication infrastructure. Ongoing monitoring and transparent reporting are therefore important to support informed decision-making and to ensure the continued protection of public health and the environment.

## Data Availability

No datasets were generated or analysed during the current study.
